# Differential Functioning of Retrieval/Comparison Processing in Detection of the Presence and Absence of Change

**DOI:** 10.1371/journal.pone.0068789

**Published:** 2013-06-24

**Authors:** Takuma Murakoshi, Masako Hisa, Yuji Wada, Yoshihisa Osada

**Affiliations:** 1 National Food Research Institute, National Agriculture and Food Research Organization, Tsukuba-shi, Ibaraki, Japan; 2 Department of Psychology, Rikkyo University, Niiza-shi, Saitama, Japan; University of London, United Kingdom

## Abstract

**Background:**

The phenomenon of change blindness may reflect the failure to detect the *presence* of change or the *absence* of change. Although performing the latter is considered more difficult than the former, the differential functioning of retrieval/comparison processing that leads to differences between the detection of the presence and the absence of change has not been clarified. This study aimed to fill this research gap by comparing performance in the detection of the presence and the absence of a change in one item among a set of items.

**Methodology/Principal Findings:**

Twenty subjects performed two types of change detection tasks, the first task was detection of one changed item among a set of unchanged items (detection of the presence of a change) and the other was the detection of one unchanged item among a set of changed items (detection of the absence of a change). The ANOVA results for the percentage of correct responses and signal detection measurement of *A’* values regarding change detection and the pattern of the results indicate that the subjects found (1) detection of the presence of change less difficult than detection of the absence of change (2), rejection of the presence of change less difficult than acceptance of the presence of change, and (3) rejection of the absence of change as difficult as acceptance of the absence of change.

**Conclusions/Significance:**

Retrieval/comparison processing for the detection of the presence of change differs from that for the absence of change, likely because the retrieval/comparison process appears aimed at determining whether an item has changed but not whether an item appears the same as it had previously. This conclusion suggests the existence of an identification process that recognizes each item as the same as that observed previously that exists apart from the mechanism underlying retrieval/comparison processing.

## Introduction

C*hange* is the transformation over time of a well defined, enduring structure [1]. Based on this definition, Simons and Rensink defined *change blindness* as the striking failure to detect a large change that would typically be detected easily [2]. Thus an individual experiencing change blindness fails to detect that an item has undergone change, perceiving the item as maintaining a well-defined, enduring structure, that is, perceiving the item to be the same as it had previously appeared. It might be assumed that the phenomenon of change blindness reflects the failure to detect the *presence* of change or the *absence* of change. In several studies, Rensink found that detecting the absence of change is much more difficult than detecting the presence of change, making the individual prone to perceiving an unchanged item as a changed item [1,3–5]. This finding implies that in the detection of the presence of change, individuals easily make the mistake of perceiving a changed item to be the same as it had previously appeared, but also easily make the mistake of perceiving an unchanged item as a changed item in the detection of the absence of change. Such competing implications suggest that different forms of perceptual processing underlie these two types of change detection. Despite such findings, as well as the knowledge that systematic comparison of the two types of detection is necessary to identify the differential perceptual processing employed in detection of the presence and the absence of change, little research has focused on the perceptual processing underlying detection of the absence of change.

Some works on the perceptual processing of subjects experiencing change blindness reported that even when subjects do not perceive a change, they retain representations of the scenes before and after the change [6–8]. This finding indicates that detection failure or change blindness may be attributable to a failure to retrieve or compare scenes before or after a change, suggesting that differences in detection of the presence and the absence of change may be due to differences in the retrieval/comparison process employed in these two types of detection [9–11]. In the natural environment, rapid detection of change is required for the identification of both dangerous entities (e.g., predators) and beneficial objects (e.g., food and water). Thus, it is plausible to assume that the change detection mechanism would place greater weight on the detection of the presence of change than the absence of change, as a lack of change would not be of interest in this context. Acceptance of such an assumption implies that retrieval/comparison processing for the detection of the presence of a change differs from that for the detection of the absence of a change. This, taken together, implies that the presence of change may be anomalous information for the visual system and that it may be processed preferentially regardless of the circumstance of that change such as what change occurred or how the change was made. If so, global information about the presence of change takes priority over detailed identification of features such as the shape or color of each item. To investigate this possibility, this study examined the nature of the detection of the presence and absence of change by using the same items but interchanging the target and distractors between both detection tasks. Static displays of both detection tasks were identical, but global dynamic information about the presence of change between displays was different. Because some top-down factors, such as a subject’s interest, knowledge or expectations, also affect the detection of change [11,12], we used the same display and varying instructions in order to exclude potential effects of top-down strategies by subjects, thus testing the intrinsic effects of the properties of stimuli. It was supposed that as set size increased, retrieval/comparison processing of each item would also increase, resulting in different retrieval/comparison load effects on performance in the detection of change with different set sizes. If the retrieval/comparison processes are different for the detection of the presence and of the absence of change, subject performance should differ with set size. We selected the set sizes based on consideration of the difficulty of both detection tasks. Previous research has shown that detection of the absence of change is more difficult than detection of the presence of change [1,3–5]. When we tested both detection tasks using the same set sizes in the pilot study, we found that the detection of the absence of change was too difficult to perform, and in contrast, the detection of the presence of change was too easy to allow for an investigation of changes in performance. Nevertheless, the use of the same set sizes in both experiments, if it had been possible, would have made the results easier to navigate. For example, presentation time could have been manipulated to vary task difficulty, allowing the use of the same set sizes in both tasks. Doing so, however, would have introduced the additional factor of presentation time, which would have prevented a direct comparison of the results of the two tasks.

## Materials and Methods

### Ethics statement

Written consent was obtained from each participant prior to experiments. The experiments were approved by the local ethics committee of the Department of Psychology of Rikkyo University.

### Experiment 1

#### Aim

Experiments 1A and 1B examined change detection by analysis of the performance of tasks using a 1-shot paradigm. In Experiment 1A, the subjects were instructed to report whether they detected a change in one item (i.e., to detect the presence of a change) in 2 sequential displays of a set of items. In Experiment 1B, they were instructed to report whether all the items were presented in the same manner (i.e., whether any of the items had changed) in two sequential displays of a set of items. Thus, the subjects had to report a change in one item in Experiment 1A, but had to report whether the entire set of items was unchanged in Experiment 1B. The aim of this manipulation of focus via providing different sets of instructions was to determine whether a difference in attentional focus would lead to differences in search strategy. By exploring the influence of search strategy, it was possible to determine the intrinsic effects of stimulus properties in a manner independent from the influence of the observer’s search strategy.

#### Subjects

All 20 participants were students at Rikkyo University (mean age = 22.02 years), participated in both Experiment 1A & 1B, had corrected-to-normal visual acuity, normal color vision, and no history of neurological problems.

#### Stimuli

**Figure 1:**
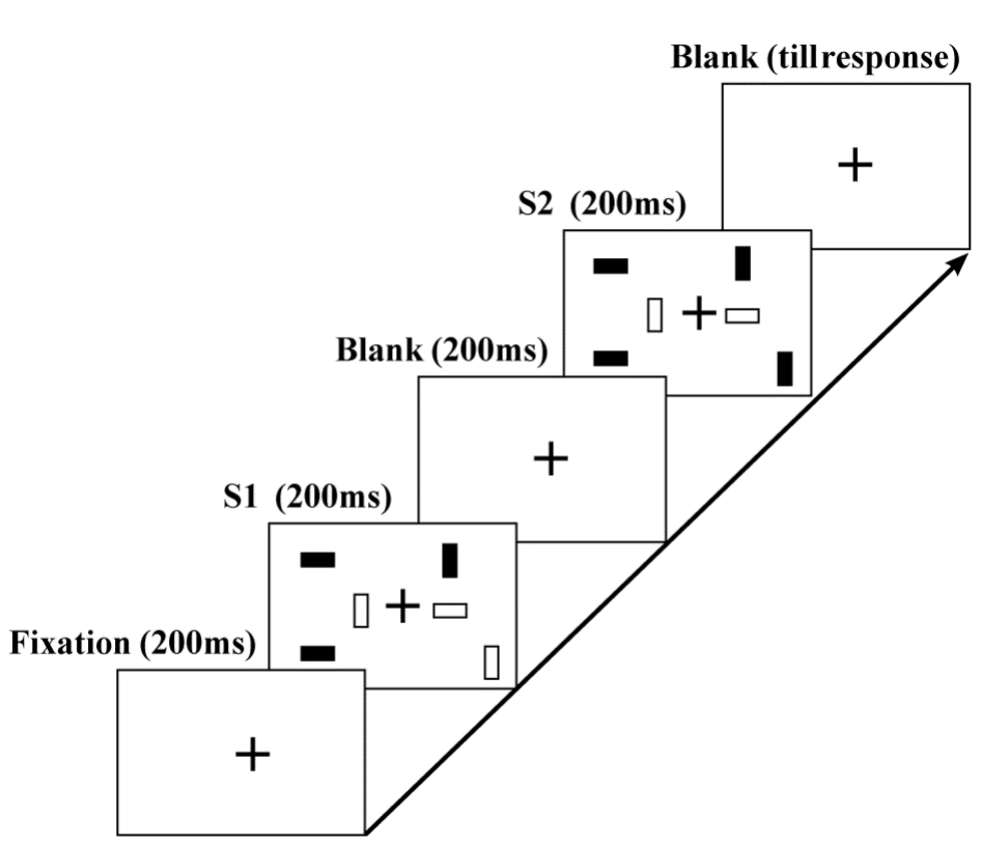
General design of the displays in Experiment 1A and 1B. The figure presents an example of a trial in which 1 of 6 presented items (i.e., a set size of 6) changed between S1 and S2.

The subjects were instructed to observe three basic types of displays, a fixation display, a test display (S1) and a comparison display (S2), presented on a video graphics array (VGA) monitor with a gray background (37.2 cd/m^2^). Examples of each type of display, along with the sequence of presentation, are shown in Figure 1. The stimuli presented in the displays consisted of a continuously visible white fixation cross (85.4 cd/m^2^) within a bounded search area. The fixation display consisted of the white fixation cross subtending at a 0.7° x 0.7° visual angle from a viewing distance of approximately 57 cm, a white outlined square centered on the fixation cross and subtending 7.4° x 7.4°, and a line 0.2° wide indicating the boundary of the search area.

#### Instruments

A Dell OPTIPLEX GX270 computer and a Cambridge Research Systems ViSaGe stimulus generator were used to run the experimental program and stimulus presentation, and a Sony GDM-F500 CRT, a chin rest, and a forehead rest were used to present the displays to the subjects.

#### Procedure

The subjects sat approximately 57 cm from the display screen in a dark room with their head in the chin and forehead rests. After they pressed a key to initiate the trial, a random interval of time (500 ms, 750 ms, or 1000 ms) passed before the fixation display was presented for 200 ms. The test display (S1) was then presented for 200 ms before the fixation display reappeared for 200 ms (a blank screen), after which the compare display (S2) was presented for 200 ms before the fixation display reappeared for a second time (Figure 1).

The S1 display consisted of the fixation display with the addition of 6, 10, or 18 bars. Each bar subtended at a 0.2° x 0.7° visual angle and was placed inside the search area such that it was located at a 1°, 2°, or 3° angle from the fixation cross both vertically and horizontally and randomly appeared as either red (20.0 cd/m^2^) or green (20.0 cd/m^2^) in color. The S2 display was identical to the S1 display except that the color of one of the bars changed from red to green or vice versa in half the trials. In Experiment 1A, the subjects were instructed to report whether they had observed a change between S1 and S2 by pressing a key on the numerical keypad. In Experiment 1B, they were instructed to report whether all the items appeared the same between S1 and S2. Thus, while identical displays were presented in Experiment 1A and 1B, the instructions presented to the subjects differed. Each subject completed 3 conditions (the presentation of 6, 10, or 18 objects) that each consisted of 50 trials, and thus completed a total of 150 trials. Prior to the experiment, all subjects completed 10 practice trials, with the order of conditions and experiments randomized for each subject.

### Experiment 2

#### Aim

Experiments 2A and 2B examined the detection of *the absence of a change*, the term used by Rensink to refer to detection of one unchanged item remaining among a set of changed objects [1,3–5]. To examine the differential nature of retrieval/comparison processing in the detection of the presence and the absence of a change, the subjects were instructed to report whether they observed an unchanged item among a set of changed items in Experiment 2, and the results were compared to those of Experiment 1. If the perceptual processing used for the detection of the presence of change differed from that used for the detection of the absence of change, different patterns would appear in the results of Experiments 1 and 2. As the same procedures were used in Experiments 1A and 2A and in Experiments 1B and 2B, differences would be attributable to differences in the properties of the stimuli presented in each experiment.

#### Subjects

The same participants who participated in Experiment 1A & 1B participated in Experiment 2A & 2B.

#### Stimuli

The stimuli used in Experiment 2A & 2B were the same as those used in Experiment 1A & 1B.

#### Instruments

The instruments used in Experiment 2A & 2B were the same as those used in Experiment 1A & 1B.

#### Procedure

The set of displays presented in Experiment 2A and 2B was the same as that used in Experiment 1A and 1B with two exceptions: (1) while the color of one item changed and the color of the other items did not change in half of the trials in Experiment 1A and 1B, the color of one item did not change and the color of other stimuli did change in half of the trials in Experiment 2A and 2B and (2) the set sizes of the items in Experiment 2A and 2B were 6, 8, and 10, but they were 6, 10, or 16 in Experiment 1A and 1B. The subjects were also presented with different instructions in each experiment. In Experiment 2A, the subjects were instructed to report whether there was one item that had *not* changed between S1 and S2 (i.e., detection of the *absence* of a change) whereas they were instructed to report whether there was one item that had *changed* (i.e., detection of the *presence* of a change) in Experiment 1A. In Experiment 2B, the subjects were instructed to report whether or not *all* of the items had changed, and were thus required to focus attention on the entire display rather than on only one item.

## Results

### Experiment 1

**Figure 2:**
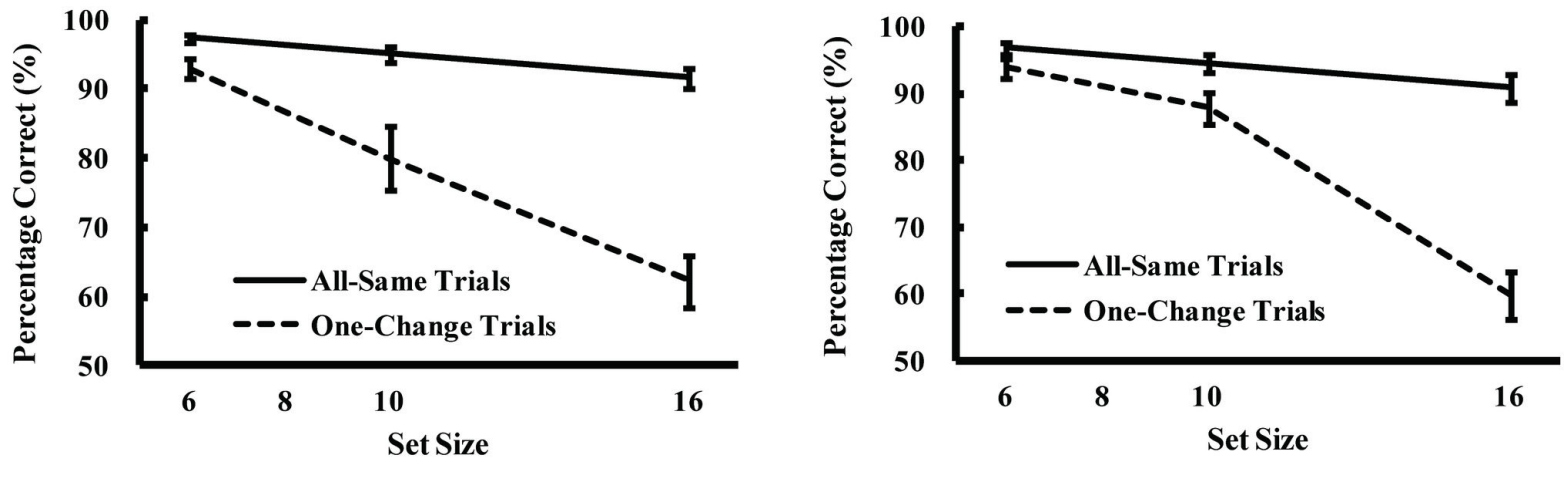
Percentage of correct responses regarding change detection plotted against set size for one-change trials (dashed lines) and all-same trials (solid lines) in Experiment 1A (Figure A) and 1B (Figure B). Error bars show standard error.

The results of error trials in which the subject pressed an irrelevant key were excluded from the analyses of Experiment 1A and 1B. The dashed lines in Figure 2A and 2B show the percentage of correct responses regarding change detection plotted against the set size for the trials in which one of the items had changed (the one-change trials) while the solid lines show the percentage correct responses for the trials in which none of the items changed (the all-same trials). As can be observed, the percentage of correct responses significantly decreased as the set size increased in the one-change trials (dashed lines), but only slightly decreased in the all-same trials (solid lines).

Examination of significant main effects with a 3-way ANOVA of the variables of experiment number (Experiment 1A or 1B), trial type (one-change or all-same), and set size (6, 10, or 16) identified the existence of a significant main effect of trial type (*F*[1,19] = 51.68, *p* < .01) and set size (*F*[2,38] = 87.73, *p* < .01), and a trial-type/set-size interaction (*F*[2,38] = 28.43, *p* < .01), but no significant main effect of experiment (*F*[1,19] = .31, *n.s.*). Examination of the simple effects of trial type for each set size identified a significantly lower percentage of correct responses in the one-change trials compared to the all-same trials for all set sizes (*F*[1,19] = 9.67, *p* < .01 for set size 6, F [1,19] = 25.37, *p* < .01 for set size 10, and *F*[1,19] = 45.53, *p* < .01 for set-size 16).

### Experiment 2

**Figure 3:**
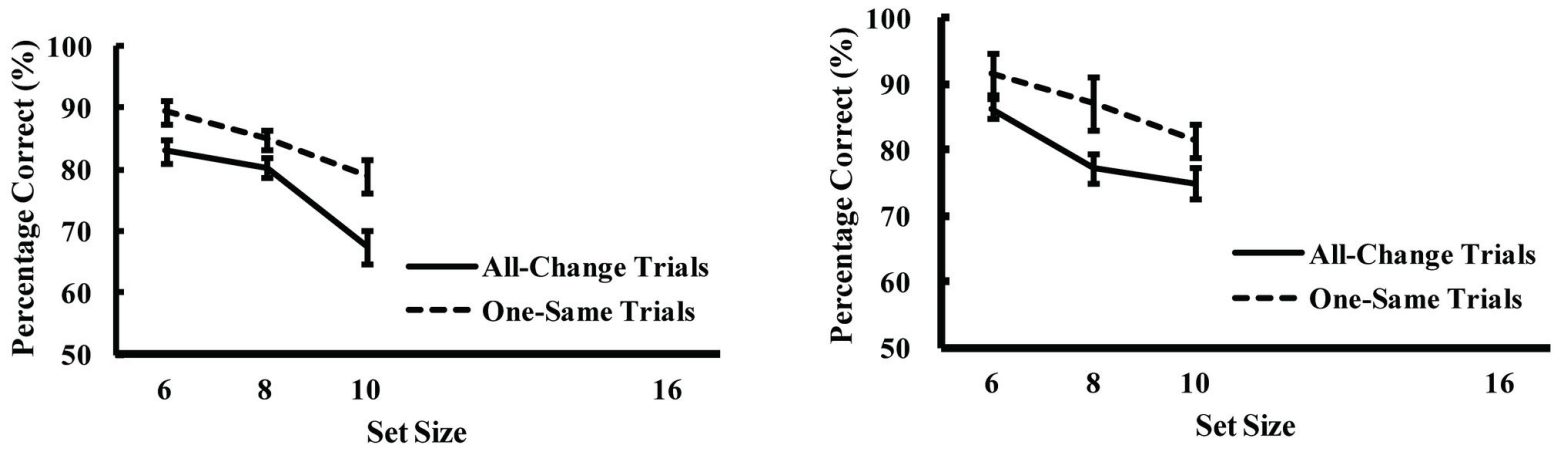
Percentage of correct responses regarding change detection plotted against set size for one-same trials (dashed lines) and all-change trials (solid lines) in Experiment 2A (Figure A) and 2B (Figure B). Error bars show standard error.

The results of error trials in which the subject pressed an irrelevant key were excluded from the analyses of Experiment 2A and 2B. The dashed lines in Figure 3A and 3B show the percentage of correct responses regarding change detection plotted against the set size for the one-same trials and the solid lines show the percentage correct responses for the all-change trials. As can be observed, the percentage of correct responses decreased as the set size increased to a similar extent in both the one-same and all-change trials.

Examination of significant main effects with a 3-way ANOVA of the results of Experiment 2A and 2B on the variables of experiment number (Experiment 2A or 2B), trial type (one-same or all-change) and set size (6, 8, or 10) identified the existence of a significant main effect of trial type (*F*[1,19] = 14.96, *p* < .01) and set size (*F*[2,38] = 31.50, *p* < .01) but not of experiment number (*F*[1,19] = 2.37, *n.s.*), and there were no interactions.

A comparison of the detection of the presence and absence of change using a 3-way ANOVA of the results of Experiments 1A and 2A on the variables of experiment number (Experiment 1A or 2A), trial type (target-present or target-absent), and set size (6 or 10) identified the existence of a significant 3-way interaction among experiment, set size, and trial type (*F*[1,19] = 5.20, *p* < .05), indicating that the effect of set size on the difference in performance across the two trial types was stronger in Experiment 1 than in Experiment 2. Post-hoc comparison revealed that in target-present trials there were no differences between percentage correct in Experiments 1 and 2 for both set sizes (6 and 10), but that in target-absent trials percentage correct in Experiment 1 was higher than that in Experiment 2 for both set sizes (6 and 10; *p* <.01 for both set sizes), indicating that the percentage of correct responses significantly differed between the two trial types in Experiment 1A (Figure 2) but did not significantly differ in Experiment 2A (Figure 3). Post-hoc comparison also revealed that percentage correct in a set size of 6 was higher than that in a set size of 10 for all conditions (*p* < .05 for target-present and target-absent trials in Experiment 1, *p* <.01 for target-present and target-absent trials in Experiment 2), indicating that none of the functions were flat, even in the all-same trials in Experiment 1A (Figure 2).

**Figure 4:**
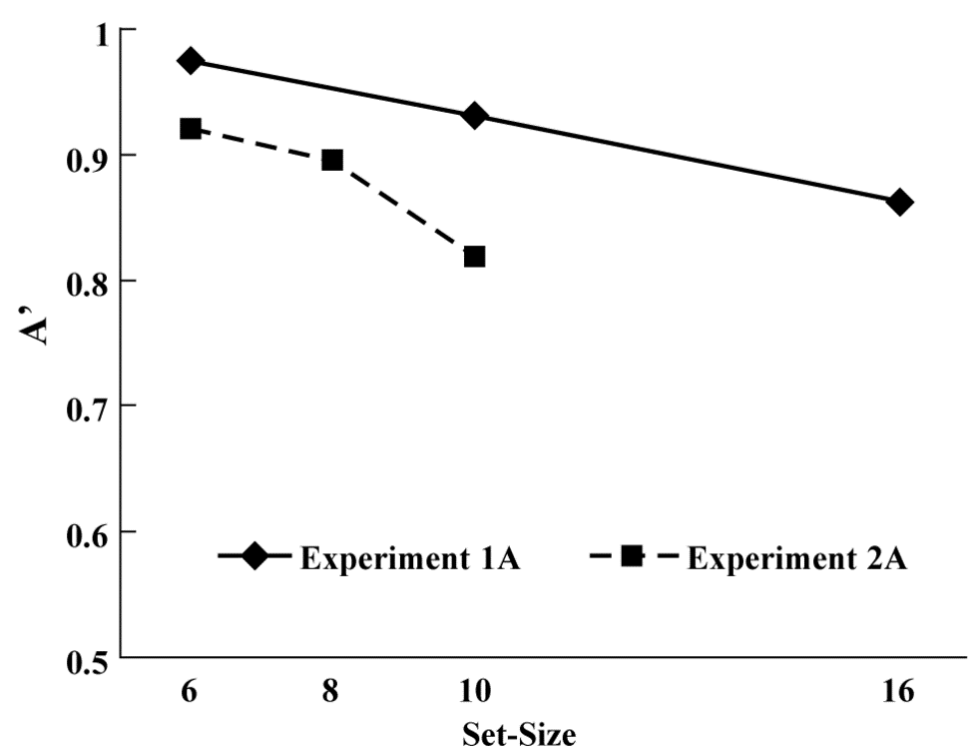
Signal detection measurement of *A* *’* values in Experiments 1A and 2A.

Figure 4 shows that in the signal detection measurement of the *A’* values in Experiments 1A and 2A, the *A’* values of Experiment 1A were higher than those of Experiment 2A in both set sizes of 6 and 10. Examination of significant effects with a 2-way ANOVA of the variables of experiment number (Experiment 1A or 2A) and set size (6 or 10) on the *A’* values in Experiments 1A and 2A identified the existence of a significant main effect of experiment (*F*[1,19] = 26.68, *p* < .01) and set size (*F*[1,19] = 26.35, *p* < .01).

## Discussion

### Experiment 1

In Experiment 1A and 1B, a significant difference was identified in the percentage of correct responses regarding change detection between the two trial types examined, specifically a lower percentage in the one-change trials relative to the all-same trials. This finding suggests that individuals are more likely to reject the presence of a change than to accept it. As such, they tend to *correctly* perceive that no change has occurred to an unchanged object as well as *incorrectly* perceive that no change has occurred to a changed item, and are thus more likely to perceive visual scenes as unchanged rather than changed. Since no significant main effect of experiment number was identified, this preference cannot be attributed to a subject’s attentional set, but can be attributed to the properties of a stimulus. Such asymmetric results for the detection of the presence and absence of change might be caused by a low level, bottom-up mechanism, but not by top-down knowledge or expectations. As change blindness may be attributable to a failure to retrieve or compare scenes before and after a change [9–11], the presence of different patterns in the results of the one-change and all-same trials implies differential functioning of retrieval/comparison processing in the acceptance and rejection of the presence of change.

### Experiment 2

In contrast to Experiment 1A and 1B, no significant interaction between trial type and set size was identified in Experiment 2A and 2B. Whereas the percentage of correct responses significantly decreased as the set size increased in the one-change trials but only slightly decreased in the all-same trials in Experiment 1A and 1B, the percentage of correct responses decreased relative to set size increases in both trial types in Experiment 2A and 2B. The results of an ANOVA performed to examine this pattern identified the existence of a significant 3-way interaction among experiment number, set size, and trial type in Experiments 1A and 2A. These results indicate that both the detection of the absence of change and the rejection of the absence of change are difficult. The results of Experiment 1A and 1B indicate that individuals are more likely to reject the presence of change than to accept it, while the results of Experiment 2A and 2B indicate that individuals are just as likely to reject the presence of change as they are to accept it. In the context of this study, this indication implies that the subjects found the rejection of the absence of change as difficult as its acceptance in Experiment 2A and 2B.

The percentage correct in the one-same trials decreased as set size increased in both Experiment 2A and 2B (see Figure 3A and 3B, dashed lines), indicating that the subjects perceived all the items as changed although one item had not changed. These findings together with those that the subjects rarely perceived a change when none was present in Experiment 1A and 1B suggests that an unchanged item tends to be perceived as a changed item when it is presented among changed items. Although the subjects had been instructed to report whether there was an unchanged item in Experiment 2A, as opposed to whether all of the items had changed in Experiment 2B, the results of both experiments indicate the existence of the same pattern, and the absence of a significant effect of experiment number in Experiment 2A and 2B suggests that the pattern cannot be attributed to the subjects’ attentional set but can be attributed to the properties of the stimuli.

As can be observed in Figure 4, the signal detection measurement of *A’* values were lower in Experiment 2A than in Experiment 1A. The existence of a significant effect of experiment number indicates that the detection of the presence of a change is easier than the detection of the absence of a change, and thus the likelihood of detecting a changed item among unchanged items is higher than detecting an unchanged item among changed items. One can suppose that the use of the same set sizes in both experiments would have made the results easier to navigate. The comparison of the different *A’* values in Figure 4 shows that Experiment 2A was more difficult than Experiment 1A. Moreover, the *A’* value for the set size of 16 in Experiment 1A was higher than that for the set size of 10 in Experiment 2A, suggesting that the bigger set sizes in Experiment 1 did not make it more difficult than Experiment 2, and thus that the set sizes modulated the task difficulty appropriately.

### General Discussion

This investigation of the mechanism underlying the detection of change identified different patterns in the detection of the presence of a change and the detection of the absence of a change. Analysis of these results indicates that the detection of the presence of change is easier than the detection of the absence of change. These results are consistent with findings from similar studies, which investigated the differences between change-present and change-absent trials [13,14]. In addition, the subject’s attentional set had no effect on performance, and thus differences in performance are attributable solely to differences in the properties of the stimulus displays. In other words, the difficulties of the two types of change detection tasks were determined by an early bottom-up mechanism in the visual pathway, and its effect was robust enough not to be affected by a higher level, top-down mechanism. Although some previous studies show a top-down attentional focus with which the expectations or experience of the observer have effects on change detection performance [11,12], the differences between performance in the detection of the presence or absence of change would reflect a fundamental mechanism of the visual system that is not effected by the higher mechanism. Because detecting change in the environment is crucial for animals, it would be important that this fundamental change-detection mechanism be achieved rapidly in a low-level stage of the visual system. These findings, together with those of previous studies which found that the individual features of an item do not affect the likelihood of change detection [15] and which found that the grouping of stimuli influences the likelihood of change detection [16], indicate that differences in performance for the two types of change detection may be due to differences in the properties of a set of stimuli. Because we used the same items in each search display, differences in the difficulty of these two types of change detection are attributable to different global information between the pre-change and post-change displays of the two tasks, but not to the properties of individual items in each display. If the visual system could identify each item in a search display, performance of presence- and absence-of-change detection would have been the same because the items used in each static display were the same in both detection tasks. Hence, it appears that the visual system does not use the identity of an individual item, but rather other properties of the stimulus display to retrieve/compare changed items. Corbett and Oriet suggested that different mechanisms underlie the processes of interpreting global information about a whole set of stimuli and the individual identity of each item in a search display [17]. Other studies have also shown that global information, such as grouping factors, about stimuli disrupts change detection performance, suggesting that items in the search display are encoded based on the global organization of stimuli, not on individual items [16,18]. Hence, the differences in performance when detecting the presence or absence of change in the present study would be due to global information between pre- and post-change displays, suggesting that the visual system may use different retrieval/comparison processes in the two types of change detection or may modulate the retrieval/comparison processes based on global information about the search display.

The results of the present study show a significant interaction between trial type and set size in detecting the presence of change, whereas no such interaction was observed in detecting the absence of change. An important factor in these types of trials is that there are no changed items in the all-same trials, whereas other types of trials included a changed item in the search display. Indeed, in the detection of the presence of a change task in Experiment 1A, the percentage correct in the one-change trials decreased as the set size increased, while the percentage correct in the all-same trials remained high (Figure 2A). In contrast, in the detection of the absence of a change in Experiment 2A, the percentage correct for both types of trials decreased as set size increased (Figure 3A). These results imply that the visual system may first abstract global information for both scenes, then if there are highly salient differences in that information, retrieve and compare those items in a slow, detailed way, whereas if differences are non-salient, the retrieval/comparison process is completed in a coarse, rapid way. When a visual scene does not contain salient information, completing a rapid exploration of that scene and being ready for more salient information in a subsequent scene is sufficient for the visual system.

There are several possible mechanisms underlying this modulation of the search process. First, shortening the retrieval/comparison time for each item makes completing the search of the display more rapid. Response time measurements for detection tasks and the calculation of search time per item may help to test this possibility. Second, varying the spatial sampling range also produces a rapid retrieval/comparison process. This sampling range would be determined by a chunk of items rather than by spatial scale. In turn, perhaps the visual system retrieves/compares some items simultaneously as a chunk. The idea of chunking some items rather than varying the spatial sampling range has merit: Large change is more detectable in a wide spatial range sampling, whereas small, salient items become less detectable in a widely distributed sampling. A change in an item is more distracting to the visual system than the lack of a change, and thus changed items cannot be easily grouped as a chunk. Also, while the visual system chunks some items, salient items cannot be chunked easily, resulting in salient items being retrieved/compared in more detail, and thus more readily detected. One more possibility is that the visual system prioritizes each item and retrieves/compares items one by one according to their priority. In this case, however, localization of the change would be completed before the retrieval/comparison process. The results of several physiological studies provide some evidence for the employment of different mechanisms relating to different aspects of change perception (e.g., change localization and change identification) [19,20], so this possibility is unlikely.

Unfortunately, we did not measure reaction time in the present study, and we acknowledge that the conclusions we can draw from this work may be limited. If further studies adopt response times, rather than accuracy, as a dependent variable of interest and compare them with the same set sizes for detection of the presence/absence of change, it could help identify the different number of items that are processed within each retrieval/comparison process while detecting the presence or absence of change.

In summary, the visual system appears to modulate the retrieval/comparison process based on global information between pre- and post-change scenes. This modulation may be attributable to a detailed search for an “important” event for an animal: Such importance emerges with the presence of change, but not with the absence of change. Thus, the retrieval/comparison process works only anomalously on a changed item, leading to the perception of what change occurred and where, but not identifying how an item is the same as it was previously. When retrieval/comparison processing fails to detect a change, items judged as not having changed are not necessarily expected to be identical. Thus, to ensure conscious recognition of individual items, detection of the absence of change alone is likely insufficient, as it does not directly lead to recognition that the items are the same as those observed previously.
